# 

**Published:** 1899-10

**Authors:** 


### BOOKS RECEIVED.

Minor Surgery and Bandaging. By Henry R. Wharton, M. D., Demonstrator of Surgery in the University of Pennsylvania. New (4th) edition. In one 12mo volume of 594 pages, with 502 engravings, many being photographic. Cloth, $3.00 net. Philadelphia and New York: Lea Brothers & Co.

Schleif's Materia Medica and Therapeutics. A Manual of Materia Medica, Therapeutics, Medical Pharmacy, Prescription Writing and Medical Latin. For the use of students and practitioners of medicine. By William Schleif, Ph. G., M. D., Instructor in Pharmacy in the University of Pennsylvania. In one very handsome 12mo volume of 352 pages. Cloth, $1.50 net. Philadelphia and New York: Lea Brothers & Co.

Transactions of the Ohio State Medical Society. Fifty-fourth annual meeting, held at Springfield, May 10-12, 1899. Edited by P. Maxwell Foshay, M. D. Cleveland: J. B. Savage. 1899.

Transactions of the Medical Society of the State of New York, for the year 1899. Edited by Frederic C. Curtis, M. D., Secretary. Philadelphia: Wm. J. Dornan, Printer. 1899.

Transactions of the State Medical Society of Wisconsin, Vol. XXXIII., for the year 1899. Edited by Charles S. Sheldon, Secretary. Madison: Tracy Gibbs & Co. 1899.

Index-Catalogue of the Library of the Surgeon-General's Office, United States Army. Authors and subjects. Second series, Vol. IV. D—Emulsions. Prepared under the direction of the Surgeon-General by James C. Merrill, Major and Surgeon U. S. Army, Librarian Surgeon-General's Office. Washington: Government Printing Office. 1899.

Transactions of the South Carolina Medical Association. Forty-ninth annual meeting, held at Harris Lithia Springs, April 5, 6 and 7, 1899. Charleston, S. C.: The Daggett Printing Co., 151 and 153 East Bay. 1899.

Practical Anatomy. Including a Special Section on the Fundamental Principles of Anatomy. Edited by W. T. Eckley, M. D., Professor of Anatomy in the College of Physicians and Surgeons, University of Illinois; Professor of Anatomy in the Northwestern University Dental School; Professor of Anatomy in the Chicago Clinical School, and Director of the Chicago School of Anatomy and Physiology; Member of the American Medical Association, the Chicago Pathological Society, the Chicago Medical Society, etc.; and Mrs. Corinne Duford Eckley, Instructor of Anatomy in the Northwestern University Woman's Medical School; Professor of Anatomy in the Chicago School of Anatomy and Physiology. With 347 illustrations, many of which are in colors. Octavo. Price, cloth, $3.50 net; oil cloth, $4.00 net. Philadelphia: P. Blakiston's Son & Co., 1012 Walnut Street. 1899.

A Compend of the Diseases of the Eye and Refraction. Including treatment and surgery. By George M. Gould, A. M., M. D., and Walter L. Pyle, A. M., M. D. Second edition, revised and enlarged; 109 illustrations, several in colors. Quiz Compends No. 8. Philadelphia: P. Blakiston's Son & Co. 1899.

The Johns Hopkins Hospital Reports. Volume VII., Nos. 5-6-7-8-9. Hernia. By Joseph C. Bloodgood, M. D. Baltimore: The Johns Hopkins Press. 1899.

Eighteenth Annual Report of the State Board of Health of New York, for the year 1897. Transmitted to the legislature, February 15, 1898. In two volumes. Albany: Wynkoop-Hallenbeck-Crawford Co. 1898.

A Compend of the Practice of Medicine. By Daniel E. Hughes, M. D., Chief Resident Physician Philadelphia Hospital; Physician-in-Chief Insane Department, Philadelphia Hospital, etc. Sixth physicians' edition, thoroughly revised and enlarged, including a section on mental diseases and a very complete section on skin diseases. Philadelphia: P. Blakiston's Son & Co. 1899.

A Treatise on Surgery, by American Authors. Edited by Roswell Park, M. D., Professor of Surgery in the University of Buffalo, N. Y. New condensed edition in one royal octavo volume of 1262 pages, with 625 engravings and thirty-seven full-page plates in colors and monochrome. Cloth, $6.00 net; leather, $7.00 net. Philadelphia and New York: Lea Brothers and Co.

Coakley on the Nose and Throat. The Diagnosis and Treatment of Diseases of the Nose, Throat, Naso-Pharynx and Trachea. For the use of students and practitioners. By Cornelius G. Coakley, M. D., Professor of Laryngology in the University and Bellevue Hospital Medical College, New York. In one handsome 12mo volume of 536 pages, with ninety-two engravings and two colored plates. Cloth, $2.75 net. Philadelphia and New York: Lea Brothers & Co.

Kirke's Handbook of Physiology. Fifteenth American edition. Revision by Warren Coleman, M. D., Professor of Physiology in the Woman's Medical College, New York, etc., and Charles L. Dana, A. M., M. D., Professor of Nervous and Mental Diseases in the New York Post-Graduate Medical School, etc. One volume, 856 pages, 8vo. Profusely illustrated by 516 wood-engravings in black and colors, and by chromo-lithographic plate. Muslin, $3.00 net; leather, $3.75 net. New York: William Wood & Co.

Progressive Medicine. Volume III. A Quarterly Digest of Advances, Discoveries and Improvements in the Medical and Surgical Sciences. Edited by Hobart Amory Hare, M. D., Professor of Therapeutics and Materia Medica in the Jefferson Medical College of Philadelphia. Octavo, handsomely bound in cloth, 440 pages, eleven illustrations. Philadelphia and New York: Lea Brothers and Co.

